# The minimal clinically important difference of six-minute walk in Asian older adults

**DOI:** 10.1186/1471-2318-13-23

**Published:** 2013-03-06

**Authors:** Boon Chong Kwok, Yong Hao Pua, Kaysar Mamun, Wai Pong Wong

**Affiliations:** 1 Clinical Services (Allied Health), National Healthcare Group Polyclinics, Commonwealth Lane, Singapore, Singapore; 2 Department of Physiotherapy, Singapore General Hospital, Outram Road, Singapore, Singapore; 3 Department of Geriatric Medicine, Singapore General Hospital, Outram Road, Singapore, Singapore; 4 Academic Division, Singapore Institute of Technology, North Bridge Road, Singapore, Singapore

**Keywords:** 6-minute walk distance, Minimal clinically important difference, Older adult

## Abstract

**Background:**

Rehabilitation interventions promote functional recovery among frail older adults and little is known about the clinical significance of physical outcome measure changes. The purpose of our study is to examine the minimal clinically important difference (MCID) for the 6-minute walk distance (6MWD) among frail Asian older adults.

**Methods:**

Data from the “Evaluation of the Frails’ Fall Efficacy by Comparing Treatments” study were analyzed. Distribution-based and anchor-based methods were used to estimate the MCID of the 6MWD. Participants who completed the trial rated their perceived change of overall health on the Global Rating of Change (GROC) scale. The receiver operating characteristic curve (ROC) was used to analyze the sensitivity and specificity of the cut-off values of 6MWD (in meters) for GROC rating of “a little bit better” (+2), based on feedback from participants.

**Results:**

The mean (SD) change in 6MWD was 37.3(46.2) m among those who perceived a change (GROC ≥ 2), while those who did not was 9.3(18.2) m post-intervention (*P* = 0.011). From the anchor-based method, the MCID value for the 6MWD was 17.8 m (sensitivity 56.7% and specificity 83.3%) while distribution-based method estimated 12.9 m.

**Conclusion:**

The MCID estimate for 6MWD was 17.8 m in the moderately frail Asian older adults with a fear of falling. The results will aid the clinicians in goal setting for this patient population.

**Trial registration:**

Australian New Zealand Clinical Trials Registry number: ACTRN12610000576022

## Background

Functional decline in older adults is frequently documented and may be characterized by slow gait speed and poor physical endurance [1-3], leading to frailty and fall [4]. Researchers and clinicians therefore measure gait performance to predict, prevent and manage frailty in older adults.

Older adults with poor fall efficacy tend to have reduced mobility which, in turn, may lead to a gradual decline in ambulatory capacity [4,5]. Thus, the six-minute walk distance (6MWD) that evaluates physical endurance is evidently an important measure [6] and clinicians can use the 6MWD to set pre-intervention goals and to assess the outcome changes post-intervention. Several studies have demonstrated that rehabilitative interventions can significantly improve physical endurance [7-9], and as a corollary, reduce functional decline, frailty and fall. Researchers and clinicians, however, are faced with the challenge of interpreting these statistically significant improvements.

The concept of minimal clinically important difference (MCID) guides the interpretation of small meaningful change as a result of interventions [2,3,10-12]. Patients determine what constitutes a clinically important difference from baseline, which is the basis of “anchor-based” method in determining MCID [2,10,13] and also its advantage over other methods. In addition, a second method (distribution-based method) to derive the MCID value was recommended [13,14]. One distribution-based method is to derive the standard error of measurement (SEM), which may be representative of the MCID value [14,15].

Increasingly, the MCID values of 6MWD were being established for various disease populations. However, almost all of these were based on Western populations [12,16-18]. Patients’ perception of clinically important change could be influenced by the population sample and socio-cultural factors such as physical activity participation. We hypothesized that the 6MWD would be lower in Asian population as compared to non-Asian population. Therefore this study was undertaken to determine the MCID for 6MWD in moderately frail Asian older adults with a fear of falling.

## Method

### Participants

The study sample was derived from the randomized controlled trial “Evaluation of the Frails’ Fall Efficacy by Comparing Treatments” (EFFECT) study. The recruitment inclusion and exclusion details have been described elsewhere [19]. The study included community dwelling older adults who had a fear of falling. Significant cognitive disorder, unstable medical and surgical conditions were the main exclusion criteria. We defined “moderately frail” as having a score of 5–9 on the Short Physical Performance Battery [20]. Eighty participants were recruited in the study, seven dropped out of the study and thus data from 73 participants were analyzed (<10% drop-out). In our study, participants from the Wii and gym exercise groups had group exercise intervention duration of one hour per session over a period of 12 weeks. The detailed intervention protocol (balance, strengthening, aerobic and stretching) for each group had been previously reported [19].

The SingHealth Centralised Institutional Review Board approved this study (Reference: 2010/177/D) and written informed consent was obtained from the participants. No adverse events were reported throughout the study.

### Outcome measure

The outcome measure, 6MWD, was assessed one week before and after the 12-week intervention by an independent blinded assessor. The 6MWD was assessed according to the established guidelines for instructions [21,22], but as a 15-meter quiet corridor was not available, we used a 10-meter quiet corridor. We decided that this minor change would not drastically impact our study findings because a study found no statistical significant difference between using two different walkway lengths of 20 m and 50 m [23]. This implied that a 30 m change in walkway length would not statistically affect the test outcome and hence the use of 10-meter walkway was appropriate.

The minimal clinically important difference was defined as the smallest difference in score in the domain of interest which patients perceived as beneficial. The global rating of change (GROC) scale ranging from −7 to 7 as shown in Table 1 was administered to ascertain the amount of change that the participants felt after the intervention [24]. The outcome assessor questioned each participant during the follow-up by asking, “Overall (health), how much change do you perceive after the intervention compared to first visit?” which was in relation to the overall benefits experienced from the intervention. Depending on the literacy of the participant, this question was asked in either English or Mandarin.

**Table 1:** **Adapted GROC scale**[24]

**Overall (health), how much change do you perceive after the intervention compared to first visit?**	
A very great deal worse	(-7)
A great deal worse	(-6)
Quite a bit worse	(-5)
Moderately worse	(-4)
Somewhat worse	(-3)
A little bit worse	(-2)
A tiny bit worse	(-1)
About the same	(0)
A tiny bit better	(+1)
A little bit better	(+2)
Somewhat better	(+3)
Moderately better	(+4)
Quite a bit better	(+5)
A great deal better	(+6)
A very great deal better	(+7)

### Statistical analyses

In order to derive the MCID with the distribution-based method, we used the SEM of the 6MWD. The baseline standard deviation, SD, and test-retest reliability, intraclass correlation coefficient (ICC [1,3]), of the 6MWD among participants who did not perceive change were used to calculate the SEM [12]. The formula for deriving SEM was SD × √ (1 − ICC).

The MCID estimate from the anchor-based method was derived from the difference in pre- and post-intervention 6MWD and GROC score. The sensitivity- and specificity-based approach was used in our study to determine the MCID [25]. The receiver operating curve (ROC) was used to estimate the sensitivity and specificity of 6MWD threshold change [11,26]. The ROC curve would illustrate the rate of change in 6MWD (sensitivity) against participants’ self-perceived improvement of GROC score 2 and above (specificity). The area under curve (AUC) of the ROC determined the accuracy of the outcome measure.

Due to the lack of literature supporting the optimal cut-off on the GROC [3], we decided to focus on what would be both minimally and clinically important to the older adults. Setting the cut-off score at “a tiny bit better” (+1) might be minimally but not clinically important. Furthermore, the older adults perceived “a little bit better” to be clinically important to them. We therefore selected “a little bit better” (+2) on the GROC as the cut-off.

Fear of falling might affect ambulation status; hence it was necessary to evaluate its association with 6MWD. Spearman correlation analysis was performed to evaluate the association between changes in fear of falling and changes in 6MWD.

Statistical analysis was performed with SPSS version 18. The ROC curve was generated using R software version 2.15.2. Statistical significance was set at *P* less than 0.05.

## Results

The participants’ mean (SD) – age was 70.0 (7.2) years, body mass index 22.4 (3.9) kg/m^2^ and Short Physical Performance Battery score 7.7 (1.3). The overall participants’ demographics were shown in Table 2.

**Table 2:** Baseline characteristics (N = 73)

**Demographics**	**Values**
Age, mean (SD), years	70.0 (7.2)
Gender - Female, n (%)	62 (84.9)
Ethnic - Chinese, n (%)	71 (97.3%)
Height, mean (SD), m	1.55 (0.06)
Body mass index, mean (SD), kgm^-2^	22.4 (3.9)
Had a fall in the past 1 year – Yes, n (%)	41 (56.2%)
Number of medications per day, n (%)	
0	17 (23.3)
1	11 (15.1)
2	9 (12.3)
≥3	36 (49.3)
Number of comorbidities, n (%)	
0	6 (8.2)
1	14 (19.2)
2	21 (28.8)
≥3	32 (43.8)
SPPB score, mean (SD)	7.7 (1.3)

The 6MWD showed a general trend of improvement with interventions. Participants who have completed the study experienced a mean (SD) change in 6MWD of 35.0 (45.2) m and Table 3 showed the comparison of 6MWD between the two groups. Participants who perceived a change (GROC ≥ 2) had a greater change in 6MWD from baseline than those who did not perceive a change (GROC < 2). There was a statistical difference between the 2 groups for change in 6MWD, *P* = 0.011 (Table 3). The mean (SD) GROC rating of the participants was +4.5 (1.8), range 0 to +7.

**Table 3:** 6MWD of participants with GROC < 2 (n = 6) and GROC ≥ 2 (n = 67)

**6MWD (m)**	**Overall**	**GROC < 2**	**GROC ≥ 2**	***P *value**
Pre-intervention				
Mean (SD)	294.7 (78.4)	269.2 (93.9)	297.0 (77.3)	0.41
Post-intervention				
Mean (SD)	330.0 (77.6)	278.4 (91.9)	334.3 (75.3)	0.091
Change from baseline				
Mean (SD)	35.0 (45.2) *	9.3 (18.2)	37.3 (46.2)	0.011 ^†^

* Pre- and Post-intervention comparison, *P* < 0.001.

^†^ Analysed with Welch-*t* test.

The SEM was calculated from an intraclass correlation coefficient of 0.981 and baseline SD of 93.9 m. These values translated to a SEM of 12.9 m for the 6MWD.

**Figure 1:**
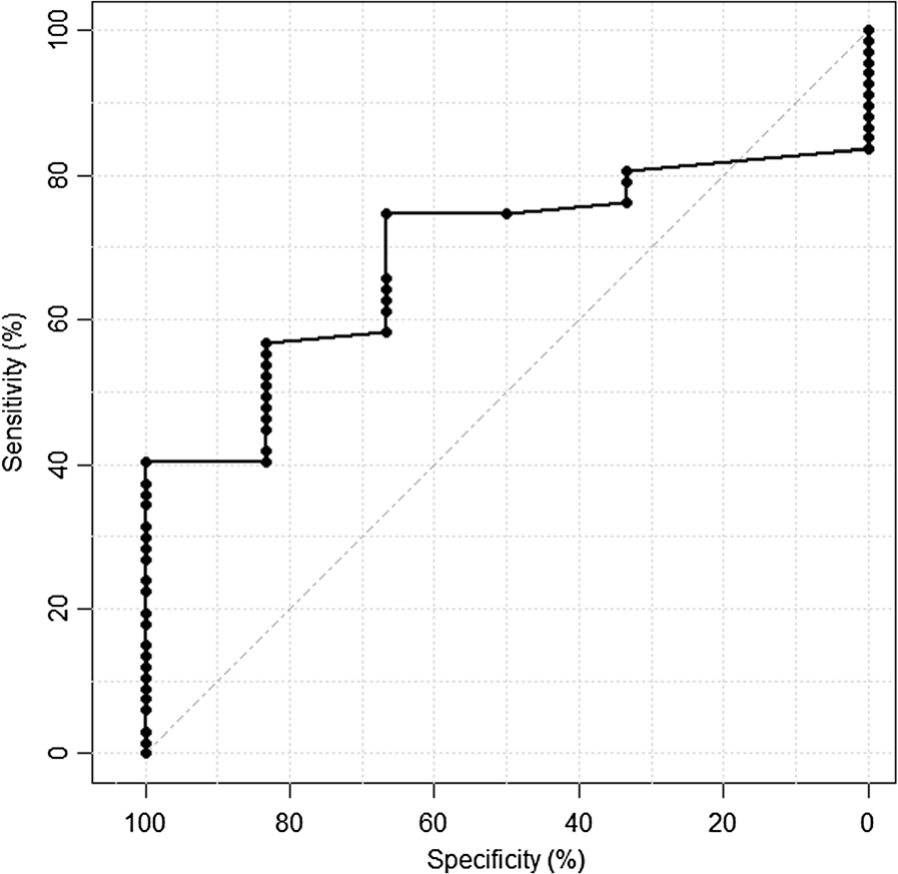
ROC curve of 6MWD.

There were 2 cut-offs identified from the ROC analysis in Figure 1, AUC 0.70, 95%-CI 0.54 to 0.84, 14.0 m (sensitivity 64.2% and specificity 66.7%) and 17.8 m (sensitivity 56.7% and specificity 83.3%). Finally, the fear of falling was weakly related to 6MWD (Spearman’s ρ 0.04, *P* = 0.75).

## Discussion

In our study of frail older adults with a fear of falling, the estimated MCID value of the 6MWD was 17.8 m. There was little difference, approximately 5 m, between the distribution-based (12.9 m) and anchor-based methods (17.8 m). Furthermore, the MCID estimate from the anchor-based method should exceed the SEM from the distribution method [12,25]. Hence, the MCID estimate of 17.8 m in this study was valid because it was above the measurement error of the 6MWD. Our study finding was aligned with the study hypothesis (smaller MCID value).

Our MCID value as compared to present literature was lower (7 to 36 m) [12,16,18], but it was comparable to that (20 m) reported by Perera et al. who studied community dwelling older adults with or without mobility decline (stroke) [17]. A recent study on heart disease population found that the MCID value was 25 m for the 6MWD [12], while the MCID value was 30.5 m for a population of pulmonary diseases [16] and a review on severe pulmonary disease population reported that MCID value was 54 m [18]. Evidently, the baseline mean of 6MWD in our study was about 300 m as compared to Holland et al’s 400 m, which could account for our smaller MCID value. Furthermore, the participant’s shorter height may be a factor of reduced 6MWD performance [22]. Hence, our MCID estimate would be proportionately smaller as compared to taller non-Asian population.

We postulate two reasons to explain the differences in findings. Firstly, the difference in population sample examined would yield different MCID values while studies examining similar population sample would yield comparable MCID values [16,17]. Secondly, socio-cultural differences might have influenced the extent of change to be meaningful. A study had found that the older Asian was less physically active compared with older non-Asian [27]; hence, it was plausible that a small change in 6MWD could be perceived as a clinically important difference. On the other hand, specific to the Singaporean context, amenities are within short commuting distances. For instance, basic amenities such as grocery shops selling daily necessities and transport stations are widely available within short distances (200 meters). Thus a small improvement in walking distance would likely be perceived as meaningful. The use of self-reported GROC as the anchor to derive MCID for 6MWD, as opposed to the use of rater-derived anchors, for example level of disability using Barthel Index, was therefore appropriate in this study and reflected the societal norm.

Because the study involved older adults with fear of falling, it was necessary to discuss the potential effects of the fear of falling on 6MWD changes which, in turn, would impact the MCID estimates. Current literature has not found an association between fear of falling and 6MWD. The present literature had identified a correlation between fear of falling and post-intervention gait speed change, but this association might not translate to other ambulatory measures [28,29]. The association between changes in fear of falling and 6MWD was poor in our study. Therefore the alleviation of fear of falling did not affect our MCID estimate.

Our study has limitations. We defined moderately frail with the SPPB score of 5–9 but other definitions of frailty exist [30]. Care must be taken when generalizing the results to the mild or severely frail individuals because their expectations and perceptions of intervention outcomes might differ. Next, our MCID value was derived from the GROC rating of a 12-week exercise intervention program. The GROC rating was based on overall change from baseline, which encompassed multiple domains – physical, function, quality of life and self-efficacy. Another limitation was the small number of participants without a GROC change, which might have affected the accuracy of estimating the specificity for the cut-off value. Also, the study was derived from a randomized controlled trial that was not primarily designed to estimate the MCID of 6MWD.

This study provides evidence that the Asian population has a smaller 6MWD MCID estimate as compared to present literature on non-Asian population. To our knowledge, this is the first 6MWD MCID established for frail Asian older adults. For the researchers, the MCID facilitates the interpretation of study findings. For the clinicians, the MCID allows quantification of rehabilitation goals. To the policy makers, the MCID helps the design of alternatives and resource allocation.

## Conclusion

A small change in 6MWD (17.8 m) was perceived as minimally and clinically important by moderately frail Asian older adults with declining fall efficacy. Further research in this area is required as there are other methods of establishing the MCID value.

## Competing interests

The authors declared that they have no competing interests.

## Authors’ contributions

BCK was principally responsible for the study design, data collection and analysis, and drafting of the manuscript. YHP was principally responsible for providing conceptual input, assisting with data analysis and interpretation and contributing to manuscript writing. KM provided intellectual inputs to the study design, participant recruitment and manuscript revision. WPW was the senior editor for the manuscript. All authors read and approved the final manuscript.

## Pre-publication history

The pre-publication history for this paper can be accessed here:

http://www.biomedcentral.com/1471-2318/13/23/prepub

## References

[B1] Hardy SE Perera S Roumani YF Chandler JM Studenski SA Improvement in usual gait speed predicts better survival in older adults J Am Geriatr Soc 2007 55 11 1727 1734 10.1111/j.1532-5415.2007.01413.x 17916121

[B2] Kwon S Perera S Pahor M Katula JA King AC Groessl EJ Studenski SA What is a meaningful change in physical performance? Findings from a clinical trial in older adults (the LIFE-P study) J Nutr Health Aging 2009 13 6 538 544 10.1007/s12603-009-0104-z 19536422 PMC3100159

[B3] Brach JS Perera S Studenski S Katz M Hall C Verghese J Meaningful change in measures of gait variability in older adults Gait Posture 2010 31 2 175 179 10.1016/j.gaitpost.2009.10.002 19889543 PMC2818277

[B4] Rantakokko M Manty M Iwarsson S Tormakangas T Leinonen R Heikkinen E Rantanen T Fear of moving outdoors and development of outdoor walking difficulty in older people J Am Geriatr Soc 2009 57 4 634 640 10.1111/j.1532-5415.2009.02180.x 19392955

[B5] Delbaere K Sturnieks DL Crombez G Lord SR Concern about falls elicits changes in gait parameters in conditions of postural threat in older people J Gerontol A Biol Sci Med Sci 2009 64 2 237 242 19196645 10.1093/gerona/gln014 PMC2655012

[B6] Rikli R Jones C The reliabiltiy and validity of a 6-minute walk test as a measure of physical endurance in older adults JAPA 1998 6 4 363 375

[B7] Leveille SG Wagner EH Davis C Grothaus L Wallace J LoGerfo M Kent D Preventing disability and managing chronic illness in frail older adults: a randomized trial of a community-based partnership with primary care J Am Geriatr Soc 1998 46 10 1191 1198 9777899 10.1111/j.1532-5415.1998.tb04533.x

[B8] Baker MK Atlantis E Fiatarone Singh MA Multi-modal exercise programs for older adults Age Ageing 2007 36 4 375 381 10.1093/ageing/afm054 17537741

[B9] Nelson ME Layne JE Bernstein MJ Nuernberger A Castaneda C Kaliton D Hausdorff J Judge JO Buchner DM Roubenoff R The effects of multidimensional home-based exercise on functional performance in elderly people J Gerontol A Biol Sci Med Sci 2004 59 2 154 160 10.1093/gerona/59.2.M154 14999030

[B10] Guyatt GH Osoba D Wu AW Wyrwich KW Norman GR Methods to explain the clinical significance of health status measures Mayo Clin Proc 2002 77 4 371 383 10.4065/77.4.371 11936935

[B11] Stratford PW Binkley J Solomon P Finch E Gill C Moreland J Defining the minimum level of detectable change for the Roland-Morris questionnaire Phys Ther 1996 76 4 359 365 discussion 366–358 8606899 10.1093/ptj/76.4.359

[B12] Gremeaux V Troisgros O Benaim S Hannequin A Laurent Y Casillas JM Benaim C Determining the minimal clinically important difference for the six-minute walk test and the 200-meter fast-walk test during cardiac rehabilitation program in coronary artery disease patients after acute coronary syndrome Arch Phys Med Rehabil 2011 92 4 611 619 10.1016/j.apmr.2010.11.023 21440707

[B13] Crosby RD Kolotkin RL Williams GR Defining clinically meaningful change in health-related quality of life J Clin Epidemiol 2003 56 5 395 407 10.1016/S0895-4356(03)00044-1 12812812

[B14] Wyrwich KW Minimal important difference thresholds and the standard error of measurement: is there a connection? J Biopharm Stat 2004 14 1 97 110 10.1081/BIP-120028508 15027502

[B15] Rejas J Pardo A Ruiz MA Standard error of measurement as a valid alternative to minimally important difference for evaluating the magnitude of changes in patient-reported outcomes measures J Clin Epidemiol 2008 61 4 350 356 10.1016/j.jclinepi.2007.05.011 18313559

[B16] Holland AE Hill CJ Conron M Munro P McDonald CF Small changes in six-minute walk distance are important in diffuse parenchymal lung disease Respir Med 2009 103 10 1430 1435 10.1016/j.rmed.2009.04.024 19477109

[B17] Perera S Mody SH Woodman RC Studenski SA Meaningful change and responsiveness in common physical performance measures in older adults J Am Geriatr Soc 2006 54 5 743 749 10.1111/j.1532-5415.2006.00701.x 16696738

[B18] Wise RA Brown CD Minimal clinically important differences in the six-minute walk test and the incremental shuttle walking test COPD 2005 2 1 125 129 10.1081/COPD-200050527 17136972

[B19] Kwok BC Mamun K Chandran M Wong CH Evaluation of the Frails' Fall Efficacy by Comparing Treatments (EFFECT) on reducing fall and fear of fall in moderately frail older adults: study protocol for a randomised control trial Trials 2011 12 155 10.1186/1745-6215-12-155 21682909 PMC3141531

[B20] Guralnik JM Simonsick EM Ferrucci L Glynn RJ Berkman LF Blazer DG Scherr PA Wallace RB A short physical performance battery assessing lower extremity function: association with self-reported disability and prediction of mortality and nursing home admission J Gerontol 1994 49 2 M85 M94 10.1093/geronj/49.2.M85 8126356

[B21] Guralnik JM Seeman TE Tinetti ME Nevitt MC Berkman LF Validation and use of performance measures of functioning in a non-disabled older population: MacArthur studies of successful aging Aging (Milano) 1994 6 6 410 419 7748914 10.1007/BF03324272

[B22] ATS statement: guidelines for the six-minute walk test Am J Respir Crit Care Med 2002 166 1 111 117 12091180 10.1164/ajrccm.166.1.at1102

[B23] Sciurba F Criner GJ Lee SM Mohsenifar Z Shade D Slivka W Wise RA Six-minute walk distance in chronic obstructive pulmonary disease: reproducibility and effect of walking course layout and length Am J Respir Crit Care Med 2003 167 11 1522 1527 10.1164/rccm.200203-166OC 12615634

[B24] Jaeschke R Singer J Guyatt GH Measurement of health status. Ascertaining the minimal clinically important difference Contr Clin Trials 1989 10 4 407 415 10.1016/0197-2456(89)90005-6 2691207

[B25] Copay AG Subach BR Glassman SD Polly DW Jr Schuler TC Understanding the minimum clinically important difference: a review of concepts and methods Spine J 2007 7 5 541 546 10.1016/j.spinee.2007.01.008 17448732

[B26] Wells G Beaton D Shea B Boers M Simon L Strand V Brooks P Tugwell P Minimal clinically important differences: review of methods J Rheumatol 2001 28 2 406 412 11246688

[B27] Fischbacher CM Hunt S Alexander L How physically active are South Asians in the United Kingdom? A literature review J Public Health (Oxf) 2004 26 3 250 258 10.1093/pubmed/fdh158 15454592

[B28] Liu-Ambrose T Davis JC Nagamatsu LS Hsu CL Katarynych LA Khan KM Changes in executive functions and self-efficacy are independently associated with improved usual gait speed in older women BMC Geriatr 2010 10 25 10.1186/1471-2318-10-25 20482830 PMC2887871

[B29] Rochat S Bula CJ Martin E Seematter-Bagnoud L Karmaniola A Aminian K Piot-Ziegler C Santos-Eggimann B What is the relationship between fear of falling and gait in well-functioning older persons aged 65 to 70 years? Arch Phys Med Rehabil 2010 91 6 879 884 10.1016/j.apmr.2010.03.005 20510978

[B30] Rockwood K What would make a definition of frailty successful? Age Ageing 2005 34 5 432 434 10.1093/ageing/afi146 16107450

